# VHH Nanobody Versatility against Pentameric Ligand-Gated Ion Channels

**DOI:** 10.1021/acs.jmedchem.4c00231

**Published:** 2024-06-03

**Authors:** Dorota Nemecz, Weronika A. Nowak, Ákos Nemecz

**Affiliations:** † Biochemistry Department, Nicolaus Copernicus University in Torun, 87-100 Torun, Poland

## Abstract

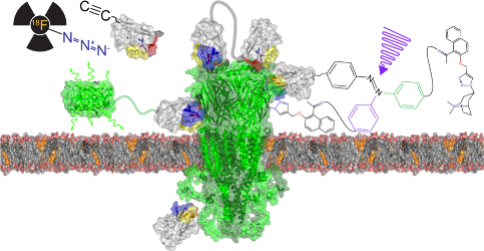

Pentameric ligand-gated ion channels provide rapid chemical–electrical signal transmission between cells in the central and peripheral nervous system. Their dysfunction is associated with many nervous system disorders. They are composed of five identical (homomeric receptors) or homologous (heteromeric receptors) subunits. VHH nanobodies, or single-chain antibodies, are the variable domain, VH_H_, of antibodies that are composed of the heavy chain only from camelids. Their unique structure results in many specific biochemical and biophysical properties that make them an excellent alternative to conventional antibodies. This Perspective explores the published VHH nanobodies which have been isolated against pentameric ligand-gated ion channel subfamilies. It outlines the genetic and chemical modifications available to alter nanobody function. An assessment of the available functional and structural data indicate that it is feasible to create therapeutic agents and impart, through their modification, a given desired modulatory effect of its target receptor for current stoichiometric-specific VHH nanobodies.

## Significance

With the discovery of heavy chain only antibodies and the powerful possibilities that their isolated variable domains reveal, there has been an explosion of their use in therapeutic research. This Perspective highlights the currently published nanobody interactions with pentameric ligand-gated ion channels, details available techniques to modify them in efforts to help develop future nanobodies as indispensable tools, furthering the understanding of these receptors and creating novel therapeutics against their associated diseases.

## 1 Introduction

Antibodies (Abs) have long been used in the study of receptors due to their high specificity and high affinity toward an antigen. However, their large size (∼150 kDa), and complex structure make their production difficult and limits their use. With the recent development and focus on single-domain antibodies (sdAbs), otherwise called nanobodies (Nbs), there is a significant potential to improve upon the capabilities that antibodies possess. Therefore, it is pertinent to discuss and identify the potential avenues of further development and the potential benefits that nanobodies may have, specifically with pentameric ligand-gated ion channels.

## 2 Pentameric Ligand-Gated Ion Channels

Pentameric ligand-gated ion channels (pLGICs) are one of the best-known family of receptors. They are ubiquitous in the central nervous system but also present in the peripheral as well as found in some non-neuronal cells. Their role is to provide rapid chemical–electrical signal transmission between cells.^1^ Human pLGICs are also known as Cys-loop receptors, after a loop in the extracellular domain, which is delimited by a pair of cysteine residues forming a disulfide bond, conserved throughout mammalian pLGICs. The family includes cation- and anion-gating channels (Table 1). Cationic channels: nicotinic acetylcholine receptors (nAChRs), the zinc-activated ion channel (ZAC), and type 3 serotonin receptors (5-HT_3_Rs), have a stimulating effect on the nervous system, whereas anionic channels: glycine receptors (GlyRs) and γ-aminobutyric acid type A receptors (GABA_A_Rs), act as inhibitory ion channels in the nervous system.^1^ PLGICs are found not only in vertebrates but also in invertebrates and prokaryotic organisms. The diversity of ligands outside of the human family increases greatly; for instance, changes in pH activate some prokaryotic pLGICs, histamine gates anionic channels in *Drosphila melanogaster*, and glutamate gates anionic channels in *Caenorhabditis elegans*.^1,2^ In addition to their endogenous ligands, pLGICs may be modulated allosterically at various locations through a plethora of either positive or negative allosteric modulators (PAMs and NAMs respectively).^3^

**Table 1:**
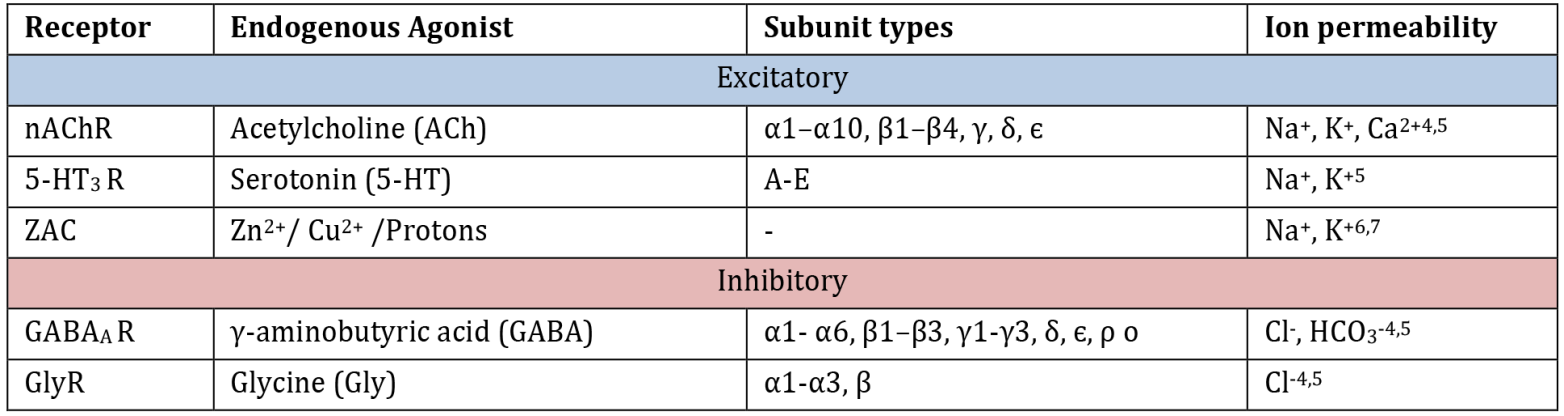
Human pLGIC Family^4−7^

**Figure 1:**
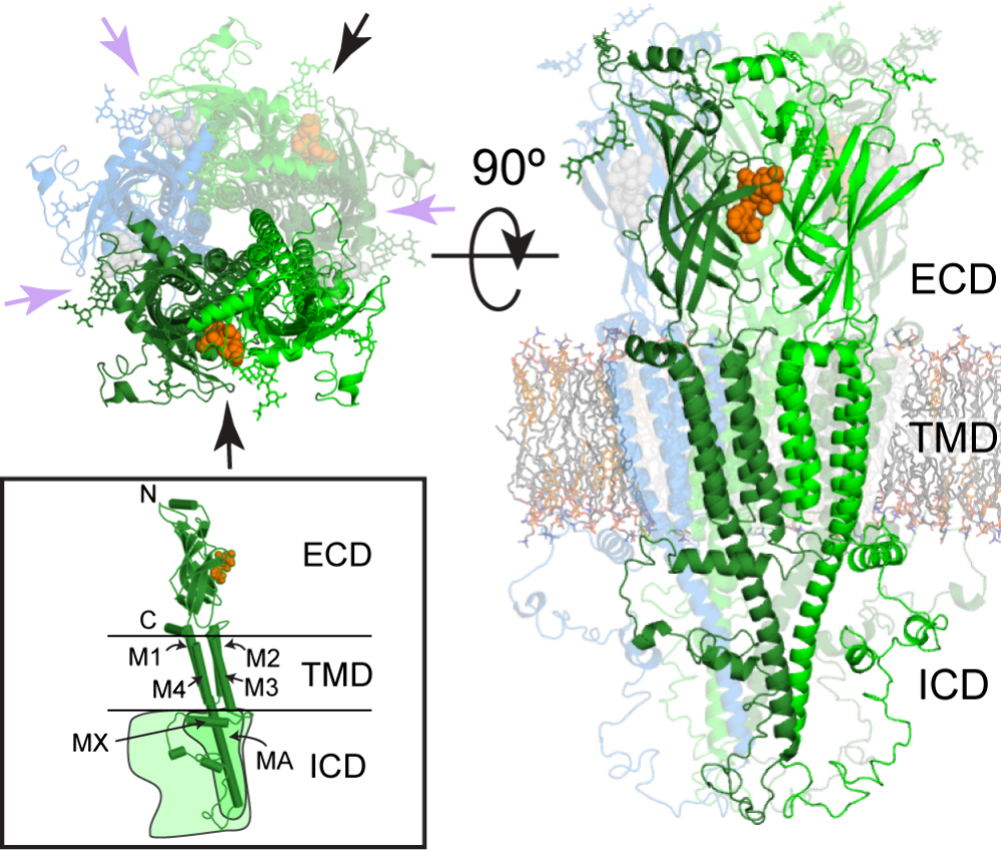
PLGIC structure. Top down (left) and side (right) view of a cartoon representation of a homomeric α7-nAChR (PDBs 7koo merged with 7rpm). Subunits are colored for a typical heteromeric receptor, where the dark-green (principal) and green (complementary) subunits compose an orthosteric binding site (black arrows and orange spheres [methyllycaconitine from PDB 3sh1]). The complementary (green) subunits in heteromeric systems need not be the same subunit. Purple arrows indicate other subunit interfaces that may act as an allosteric or different orthosteric site depending on what type of subunit occupies the blue-colored subunit. Helices are labeled in the cartoon inset depicting a single subunit, where those in the ICD are only found in cationic receptors. The two schematic outlines also depict how the size of the ICD may vary.

PLGICs are 150–300 kDa protein complexes composed of five identical (homomeric receptors) or homologous (heteromeric receptors) subunits. Subunits are arranged symmetrically around a central axis, forming an ion pore (Figure 1). Each subunit consists of an extracellular domain (ECD), a transmembrane domain (TMD) and an intracellular domain (ICD).^1^

The N-terminal ECD domain has a β-sandwich structure formed by ten β-strands (β1−β10), stabilized by conserved hydrophobic residues. It contains a ligand binding site, centrally located at the interface between two adjacent subunits (Figure 1).^1^

The TMD consists of 4 membrane-penetrating helices named M1 to M4. The M2 helix is implicated in the formation of an ionotropic pore.^1^ The M3 and M4 helices are responsible for the transport of receptors to the cell membrane, their anchoring at the synapse, intracellular interactions, and receptor phosphorylation.^8^

The ICD is located between the M3 and M4 helices, and its structure is more variable across the pLGIC family, ranging from as short as 52 residues in the ZAC, to as large as 271 residues in the α4-nAChR subunit. High-resolution structural determination of the ICD has proven to be difficult, due to its disordered nature, with exception to the ordered MX and MA helices found in the cationic channels. The resolution of the ICD using nuclear magnetic resonance of an α7-nAChR, with the ECD removed, also demonstrated the ICD’s secondary structure to be predominantly loops.^9^ The role of the ICD is primarily in regulating expression, but ICD lateral fenestrations found in cationic receptors also regulate channel activity.^10,11^

PLGICs are intensively studied on account of their role in numerous disorders of the nervous system. Disruption of nAChR and GABA_A_R expression and/or a change in their function resulting from mutations has been associated with schizophrenia.^12,13^ They have also been linked to neurodegenerative pathologies, such as Alzheimer’s disease.^14^ Mutations in pLGICs are also associated with severe congenital pathologies,^15^ including: epileptic syndromes for GABA_A_Rs,^16^ congenital myasthenic syndrome and nocturnal frontal lobe epilepsy for nAChRs,^17,18^ as well as hyperekplexia for GlyRs.^19^ Moreover, the pathological dysfunction of several pLGICs has also been linked to autism.^20−22^

## 3 Nanobodies

Since their discovery, nanobodies have revolutionized various fields, from medicine to research and diagnostics. Their unique structure and properties offer a plethora of advantages that make them an invaluable asset in diverse applications. They can be used in *in vitro* or *in vivo* imaging techniques to study localization or the interaction of specific protein or nonprotein molecules. They also have an application in protein structural and functional studies, immunoassays, and affinity purification. Additionally, nanobodies can serve as a powerful therapeutic tool, where they can act as direct activators or inhibitors of certain proteins/pathways implicated in diseases. They can even serve as a means for drug delivery. Finally, they are advantageous as a diagnostic tool for detecting marker molecules.^23^

### 3.1 Nanobody Structure

Nanobodies, or sdAbs, are the variable domain of antibodies that are composed of the heavy chain only (HCAbs). HCAbs were first discovered in 1993, in the blood of camels (*Camelus dromedarius*),^24^ whose variable domain (VH_H_) was later isolated to create sdAbs or VHHs, with a resolved structure a few years later.^25^ Today it is known that they are a characteristic feature of all animals belonging to the camelid family (*Camelidae*), which include: vicuñas, alpacas, llamas, and dromedaries.^26,27^ HCAbs that are known as immunoglobulin new antigen receptors (IgNARs) were also isolated in 1995 from nurse sharks (*Ginglymostoma cirratum*).^28^ Later IgNARs were found also in other cartilaginous fish, such as ratfish. The single chain variable domain of these antibodies is called VNAR.^27,29,30^

**Figure 2:**
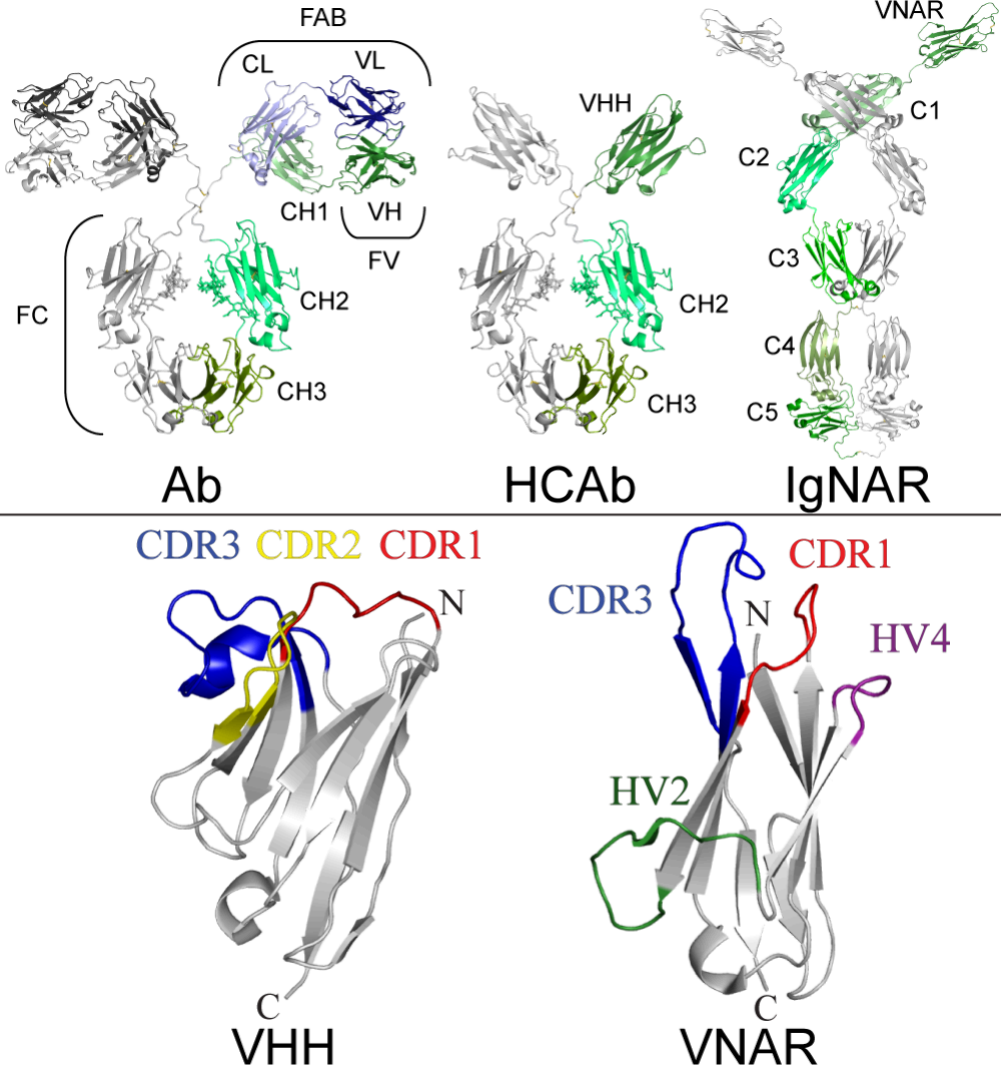
Structural comparison of antibody types and nanobodies. Top: Structures of an IgG2 class antibody (Ab, modified PDB 1igt), camelid heavy chain only antibody (HCAb, VHH from PDB 4pir and FC from PDB 1igt), and a shark immunoglobulin novel antigen receptor (IgNAR, using PDBs 4q97, 4q9b, 4q9c, 2mkl, and 2ywz). Each structure has a single heavy chain colored in shades of green with the respective domains labeled, whereas the Ab also has the respective light chain colored (blues). Bottom: Comparison between representative VHH-Nb (from PDB 6i53) and VNAR (PDB 2ywz) structures, with N- and C-terminals indicated, and the complementarity determining regions (CDRs), along with the hypervariable regions (HVs), of VNAR color coded and labeled.

Both, VHHs and VNARs, seem to be an interesting alternative to conventional antibodies. They differ structurally (Figure 2) but show similar advantages. Most research studies have, thus far, focused on using the VHH camelid sdAbs, mostly due to the easier process of immunization and blood collection.

Camelid HCAbs, unlike conventional immunoglobulins, are composed of heavy chains only. They belong to the IgG class of antibodies, specifically the IgG2 and IgG3 subclasses.^31^ HCAbs consist of a variable domain located at the N-terminus, a hinge region, and two C-terminal constant domains: CH2 and CH3 (Figure 2).^26,32^ The CH1 domain, compared to standard antibodies, is lost in the splicing process as a result of a point mutation (G to A) located in the intron preceding the CH1 domain sequence.^31,33^ The hinge region is located between the CH2 domain and the VH_H_ domain. This region is extended, compared to conventional antibodies, due to repeated Pro/Gln and Lys/Pro sequences.^33^ The hinge region on HCAbs may be classified as either a long-hinge (IgG2) or short-hinge (IgG3).^31,32^ The VH_H_ domain contains a paratope which is responsible for recognizing and attaching to the antigen.

**Figure 3:**
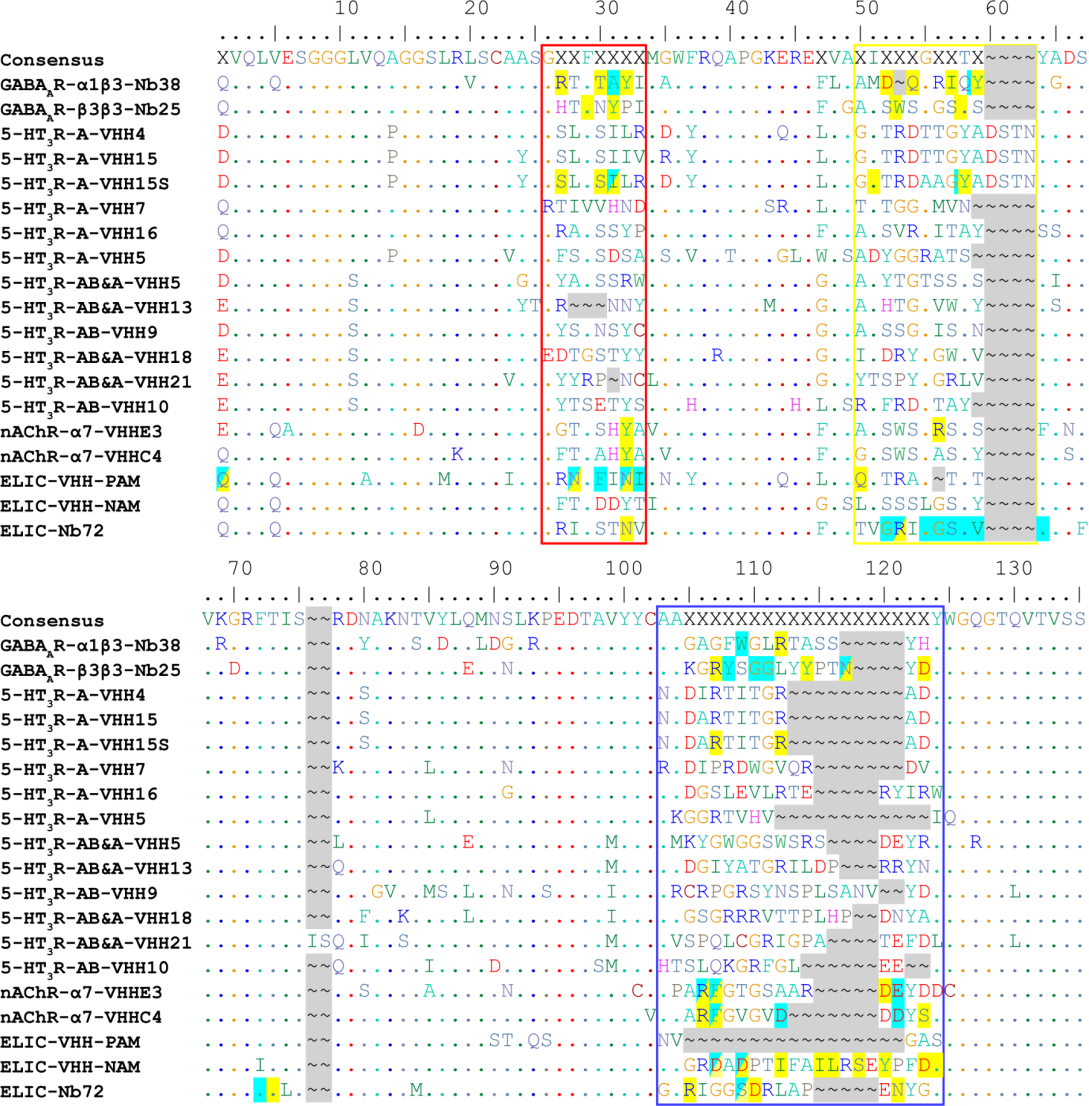
Alignment of all available pLGIC Nb sequences. For clarity, the signal peptide and C-terminal tags have been removed from all sequences. The consensus sequence represents all residues with >50% identity between the sequences with X for those residues, which are more variable. Almost all of these fall within the CDRs 1, 2, and 3, which are indicated with the red, yellow, and blue boxes, respectively. Gaps in the alignment are highlighted in gray for clarity, and residues noted in the text with direct (yellow), backbone (cyan), and mixed (half-cyan/half-yellow) interactions are also highlighted. Sequences, listed in the receptor order found in the text (with 5-HT_3_A and AB divided), have the published subunit selectivity denoted between the receptor and VHH name. PAM Nbs for each receptor are listed first, then silent or NAM Nbs in order of potency. Nb38 sequence derived from PDB 6i53 and Nb25 from PDB 5ojm. All 5-HT_3_R VHH sequences are from ref (38). Both nAChR VHH sequences are from ref (39). ELIC PAM VHH is from PDB 6ssi, ELIC NAM VHH is from PDB 6ssp, and finally ELIC Nb72 is from PDB 6hjy.

VHH Nbs are small ellipsoidal proteins (2.5 nm × 4.2 nm) with a mass of approximately 15 kDa.^26,27,31,34,35^ Within the VHH structure, four framework regions (FR1–FR4) and three hypervariable complementarity determining regions (CDR1–3) were distinguished (Figure 2).^32,34^ The FRs are composed of nine antiparallel β strands labeled A and B (FR1); C and C′ (FR2); C″, D, E, and F (FR3); and G (FR4), which form the general scaffold of the VHH.^32^ These regions show high (over 80%) homology in the amino acid sequence and spatial structure in relation to the VH domain of human antibodies.^34^ Most of the sequence differences result from somatic hypermutation of the genes encoding the VHH’s heavy chains.^33^ The FR2 contains essential changes from the conserved hydrophobic human sequence involved in the association of the light chain within conventional antibodies: (Figure 3: 5-HT_3_R-A-VHH5) Val37, Gly44, and Leu45, which are often substituted by amino acids: Phe/Tyr37, Glu44, and Arg45 (Figure 3).^31,33,34^ Such substitutions greatly increase the hydrophilicity of the VHH, and remove the association of the light chain.^33^ Additionally, some sequence variations, when compared to human antibodies, can be found in FR1 and FR3; these lie within amino acid residues 28–31 and 79–85, respectively.^33^ The hypervariable domains: CDR1, CDR2, and CDR3, have the form of loops located between the framework β strands: B and C, C′ and C′′, and F and G, respectively. They create the paratope, which is the antigen-binding surface. CDR1 and CDR3 are extended and more variable compared to conventional antibodies, which increases their surface of interaction with the antigen and compensates for the lack of the three light chain loops also involved in antigen recognition in conventional antibodies.^26,32^ Additional to the CDRs, the FR2 may also be involved in antigen binding.^31^ Moreover, in most VHHs, CDR3 connects to either CDR1 or FR2 through a disulfide bond,^34^ which increases the stiffening of the extended CDR3.^32,33^ It has been shown that this disulfide bridge is important for the thermal stability of the entire molecule but does not affect the ability to bind antigen.^36^

### 3.2 Advantages/Disadvantages of Nanobody Properties in Relation to Conventional Antibodies and Their Fragments

The unconventional structure of Nbs has many specific biochemical and biophysical properties that make them an excellent alternative to conventional antibodies. The single-chain nature makes Nbs easy to produce in prokaryotic expression systems which boast a high protein yield, compared to the modicum of conventional antibodies produced, mostly in *in vitro* cell culture (eukaryotic hosts). Even production of antibody fragments, such as Fab (fragment antigen binding) domains and scFvs (single chain variable fragments), carries numerous complications.^26^ Production of Fab fragments may result in mismatching of the light and heavy chains. Moreover, because the scFvs are fusion proteins of the VH and VL domains of standard antibodies, their proper folding is more complicated. Additionally, compared to conventional antibodies, as well as Fabs and scFvs, VHHs are more conducive to expression in the cytosolic environment, all the while maintaining antigen binding properties. Such intrabody VHHs are a great tool for studying intracellular signaling pathways.^37^

SdAbs are much easier to genetically manipulate, which allows for their direct labeling with fluorochromes, the creation of multivalent variants, or their fusion with other proteins.^34^ Such modifications increase the potential applications for which VHHs may be advantageous. The substitution of conserved hydrophobic amino acids in the FR2 ensures better solubility and prevents the aggregation of Nbs. Additionally, the hydrophilic nature and single-stranded structure make them capable of efficient refolding after denaturation. This property results in their high physicochemical stability. Studies have shown that some Nbs are thermostable and able to function even at temperatures of 90 °C.^34^ Moreover, VHHs also show a resistance to proteases activity and stability in extreme pH values.^34,35,37,40^ Due to the fact that VHHs are characterized by high homology to human VH domains, they also have low immunogenicity.

Nanobodies’ antigen affinity is comparable to conventional antibodies and is usually in the nano- or picomolar range.^40^ They can recognize and bind a wide spectrum of epitopes with high specificity. A big advantage is that their small size, as well as its extended and flexible CDR3, enable the recognition and binding of epitopes that are unrecognizable or unreachable for conventional antibodies, such as clefts and cavities.^34^

Nbs are characterized by fast blood clearance due to their small size. According to *in vivo* studies, their half-life in mice ranges from 0.5 to 2 h.^27^ This may be both an advantage and a disadvantage, depending on the planned application. A short half-life, and specifically fast blood clearance, of VHHs is beneficial for diagnostic purposes, such as *in vivo* VHH-based imaging. It leads to improved signal quality by reducing the excess unbound VHH, and thereby the background signal.^41^ Additionally, high kidney filtration limits the exposure of cells and tissues to toxic/radioactive compounds used in such techniques, preventing their negative side effects. On the other hand, a short half-life of VHHs may negatively influence the effectiveness of their use as therapeutics and/or drug delivery agents. This may arrive from a lower tumor penetration, or inability to attain the target, as a result of fast clearance. However, there are multiple ways to significantly extend a VHH’s half-life through modifications such as PEGylation (adding polyethylene glycol), and direct fusion with stable serum proteins (such as albumin or immunoglobulins) or small molecules which bind them (including bispecific VHHs).^26,40^ It has been shown that after fusion with serum proteins, the half-life of VHHs extended to the half-life of the fused protein.^34^

The small size of VHHs also allows them to penetrate tissues much better than conventional antibodies. In some cases, they can get inside the cell through the cell membrane and interact with cytosolic targets.^42^ Moreover, it has been reported that some VHHs can also cross the blood–brain barrier (BBB), profiting from the use of naturally occurring protein transporting systems such as receptor-mediated transcytosis, adsorptive-mediated endocytosis, or carrier-mediated transcytosis.^27,42,43^ These VHHs tend to have a higher pI. Some VHHs cross based on the temporary opening of the BBB in response to different environmental conditions, such as osmotic disruption or low energy ultrasounds, or in the case of some brain diseases, such as multiple sclerosis and sleeping sickness, which cause a temporary BBB opening.^27,42^ For VHHs which naturally do not cross the BBB, modifications reviewed in section 6.5 may be made to assist their crossing.

## 4 VHHs against Anionic pLGICs

### 4.1 GABA_A_R

**Figure 4:**
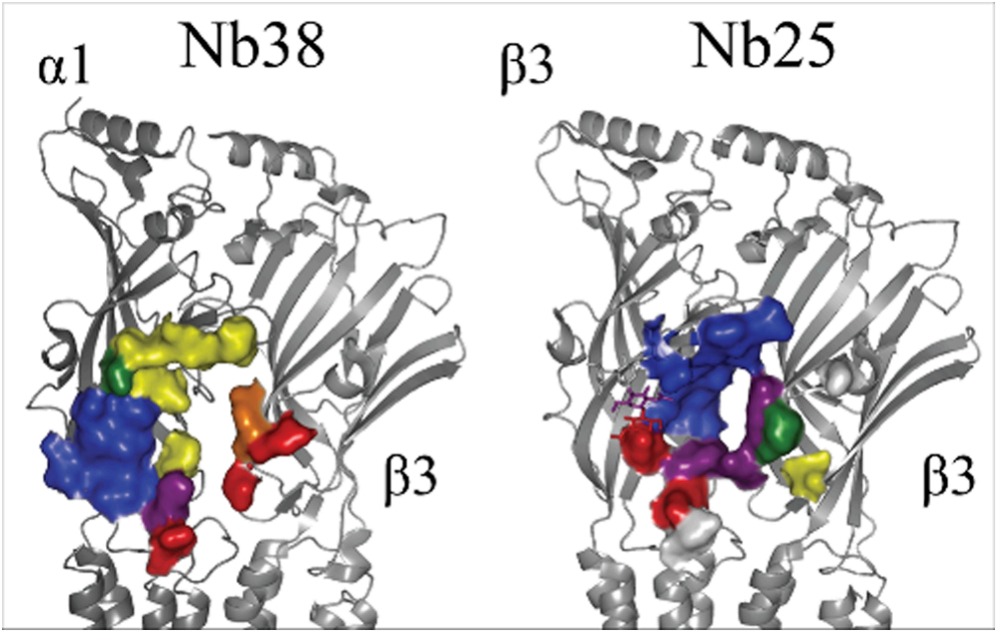
VHH binding surface on GABA_A_Rs. Side view, cartoon representation, of two subunits in a given GABA_A_R subunit-interface zoomed in on the ECD, with the bound VHH name above the structure and subunits labeled to the side. Color-coded surface representation of residues within 4 Å of the bound VHH, color-coded as follows: white for non-CDR proximity; red, CDR1; yellow, CDR2; blue, CDR3; and overlapping residue interactions as orange, CDR1 and 2; purple, CDR1 and 3; green, CDR2 and 3; and brown, CDR1 and 2 and 3. PDBs: 6i53 and 7qnb.

It was reported that a VHH library was created against purified recombinant α1β3–GABA_A_Rs with a 1D4 C-terminal tag on the α1-subunit.^44^ The selected clones from this library were divided into 13 families. Two Nbs, Nb25 and Nb38 (Figures 3 and 4), have been partially characterized in experiments in which they were used as tools to support the receptor crystallization process. Both VHHs were described as recognizing and binding the ECD of GABA_A_R (Figure 4). An unpublished preprint which is cited by subsequent publications from the same group claims that Nb38 shows high specificity toward the α1-subunit (where it does not recognize the αXβ3γ2–GABA_A_Rs [X = 2–6]) and makes specific contacts at the interface between the α1-subunit and the adjacent β3- or γ2-subunits, near the ligand binding region when using 4-fold molar excess VHH.^45^ However, a modified version of this nanobody, termed Mb38, with the inclusion of the extracellular adhesin domain of *Helicobacter pylori*, was shown to bind to the full-length α1β3γ2L–GABA_A_R, but only between the α1-β3 interface, and no binding was resolved at the α1−γ2 interface, when using 1:1 (VHH:receptor) molar ratio (PDB 6I53).^46^ Therefore, it appears that from published data Nb38 is actually selective for the α1/β3 interface.

**Figure 5:**
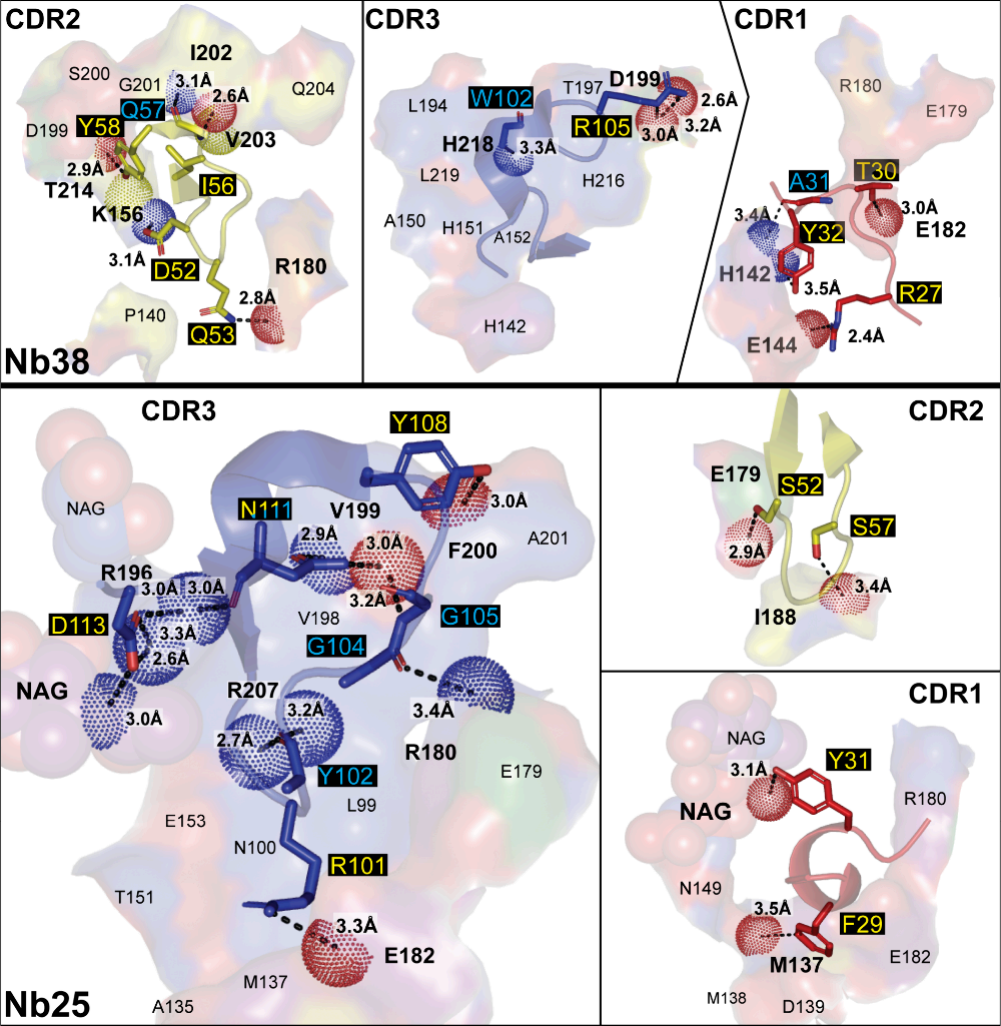
GABA_A_R VHH pharmacophore interactions. Detailed interactions of each CDR from Mb38 with the GABA_A_R α1-β3 subunit interface (top) and from Mb25 with the GABA_A_R β3−β3 subunit interface (bottom) depicted in Figure 4. CDR2 is the principal component of binding for Mb38, and CDR3 for Mb25. Each panel is labeled with its respective CDR and color coded as in Figure 4, with the exception of oxygen, nitrogen, and sulfur atoms, which are colored red, blue, and yellow, respectively. Residues within 4 Å of a CDR have their surface shown and are labeled. Residue labels in bold indicate a direct interaction with the CDR. Polar interactions are shown with black dashed lines with distances indicated. CDR residues are labeled with a black background and colored and represented as sticks according to interaction type (yellow/side chain and cyan/backbone). Acceptor, red; donor, blue; and hydrophobic, yellow; van der Waalls interacting atoms from the subunit residues are depicted as color-coded dots.

The main component of binding is the CDR2 of Mb38, sandwiched by interactions of both CDR3 and CDR1 (Figures 4 and 5). CDR2 tucks under the _α1_β9−β10 loop in the α1−β3 interface. Of CDR2, _Nb_I56 nests in a shallow hydrophobic pocket; meanwhile _Nb_D52, _Nb_Y58, and the backbone of _Nb_Q57 make polar interactions with the β9- and β10-strands of the α1-subunit and _Nb_Q53 makes a polar interaction with the backbone of _β3_R180 on the β8−β9 loop (Figure 5). CDR3’s interactions consist of a polar interaction between the backbone of _Nb_W102 and _α1_H218 on the β10-strand, with an additional salt bridge between _Nb_R105 and _α1_D199 on the β9-strand. CDR1 contributes additional interactions with the _α1_Cys-loop, where _Nb_Y32 stacks with _α1_H142 and makes a polar interaction, meanwhile the backbone carbonyl of _Nb_A31 also makes a polar interaction with _α1_H142, _Nb_R27 forms a salt bridge with _α1_E144, and _Nb_T30 makes a polar interaction with _β3_E182 on the β8-β9 loop.

Reported functional experiments conveyed that Nb38 more strongly potentiated the α1β3γ2_EM_–GABA_A_R construct than the wild-type α1β3γ2L–GABA_A_R.^45^ The affinity of both, Nb38 and Mb38, for the α1β3γ2L–GABA_A_R strongly increases with the presence of bound GABA inside the orthosteric site.^45,46^ Most interestingly, 10 μM of either version activated ∼10% of wild-type receptors, where in this case the reported activation amplitude was stronger for the wild-type over the construct used for the cryo-electron microscopy structure.^45,46^

The published structures show that Nb25 binds in the same general area as Nb38 (Figure 4), but interacting with the β3-subunit instead of the α1-subunit, specifically at β3−β3 interfaces.^44^ Specific selectivity toward this interface was confirmed by the numerous structures (PDBs: 5ojm, 7pbd, 7qn5, 7qn6, 7qnA, 7qnb, and 8pvb).^47^ Nb25’s orientation is rotated in comparison to Nb38; therefore, its CDR3 makes the principal interactions. These interactions consist of _Nb_R101, making a salt bridge with the neighboring subunit _β3_E182 on the β8−β9 loop, meanwhile _β3_R180 from the same loop interacts with _Nb_G104’s backbone carbonyl (Figure 5). _Nb_Y102’s and _Nb_G105’s backbones, along with the side chains of _Nb_Y108, _Nb_N111, and _Nb_D113, make polar contacts with the β9−β10 loop, with _Nb_D113 also having a polar interaction with the second *N*-acetylglucosamine of the sugar attached to _β3_N149. _Nb_Y31 of the CDR1 also makes a polar interaction with the first sugar. _Nb_F29 makes a weak C–H hydrogen bond with the carbonyl backbone of _β3_M137 of the Cys-loop to complete CDR1’s interactions. CDR2 makes a couple of polar interactions with the backbone carbonyls of _β3_E179, on the complementary β8−β9 loop, and _β3_I188, on the β9-strand, through _Nb_S52 and _Nb_S57, respectively. The close proximity of the CDR2 does not allow for the conformational differences in the β8−β9 loop of the other GABA_A_R subunits, where large clashes occur with all but the δ subunit, in which the existing salt bridge with the β3-subunit is lost, replacing _β3_E182 with a Q. Although binding in the same location as Nb38, it appears that the rotated orientation of Nb25 removes the PAM effect and there was no reported functional effects on either the receptor activity or even on other GABA_A_R specific PAMs.^44^

### 4.2 GlyR

Due to the ease with which the GlyR may be structurally resolved, there has not been a drive for the development of nanobodies against the receptor, as such no currently published nanobodies exist, but that does not preclude their potential as tools and selective pharmaco-agents targeting GlyRs.

## 5 VHHs against Cationic pLGICs

### 5.1 5-HT_3_R

For the 5-HT_3_Rs, it was reported that an initial library against the 5-HT_3_AR found 17 different clones. Six of these clones were subsequently described,^38^ where VHHs 15, 15S, and 7 were detailed as potent (low nM) inhibitors (15 and 15S more so than 7), meanwhile VHHs 16 and 5 were described as weaker inhibitors, giving partial inhibition and VHH5 almost fully washes out after 5 min. Conversely, VHH4 was reported to act as a PAM.

**Figure 6:**
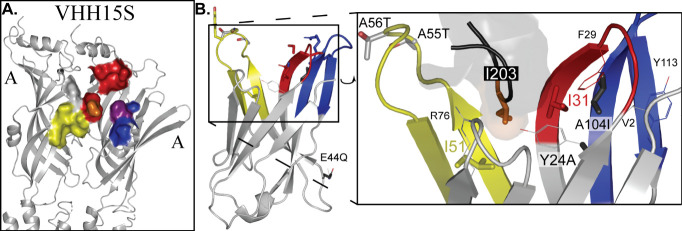
VHH binding with the 5-HT_3_AR. (A) VHH binding surface on the 5-HT_3_AR. Side view, cartoon representation of two subunits involved in the subunit–interface binding, zoomed in on the ECD, with color-coded surface representation of residues within 4 Å of bound VHH15S (following the same color code as Figure 4). PDB 4pir. (B) VHH15s vs VHH7. Interacting residues of VHH15S from (A) are shown in stick representation (removed in inset). Line (VHH15S) and stick (VHH7) representations of mutated residues are shown and labeled. Inset: Slightly rotated, for clarity, zoomed representation of I203 and the end of the β9–10 loop from the principal A subunit, with residues’ side chains within 4 Å of I203 (R76, I51, I31, and Y24) and the A104I mutation (F29, V2, and Y113) also labeled and shown in line representation.

**Figure 7:**
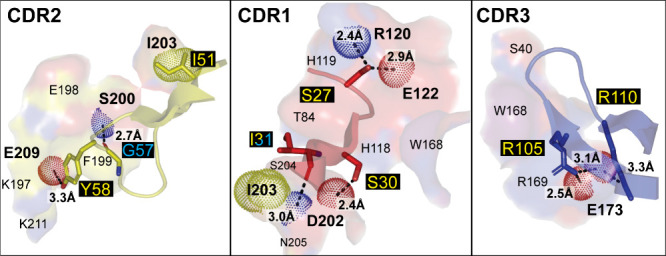
VHH15S pharmacophore interactions. Detailed interactions of each CDR from VHH15S with the 5-HT_3_AR interface depicted in Figure 6. CDR1 and 2 envelop the β9–10 loop as the principal component of binding. Each panel, labeled by its respective CDR, is depicted, color coded, and labeled as in Figure 5.

Only VHH15S was evaluated structurally (PDB 4pir),^11^ whereas VHH7 was only used in a 2D-crystal.^48^ VHH7’s binding was merely assumed to be the same as VHH15S in the modeling, and no high resolution or other form of structural localization was performed. The binding of VHH15S is located primarily on the complementary face above the ligand-binding site, where _Nb_S27 from the CDR1 make polar interactions with _A_R120, and _A_E122 of the complementary subunit’s β5−β6 loop (Figures 6A and 7). _Nb_R110 and _Nb_R105 of the CDR3 reinforces interactions with the complementary A subunit through salt bridges with _A_E173. _A_I203, from the β9−β10 loop of the principal subunit, buries deep in a hydrophobic pocket between CDRs 1 and 2 (_Nb_I31 and _Nb_I51, respectively), along with the loop between the β-sheets of FR3. _Nb_I31 reinforces the interaction with a hydrogen bond between its backbone carbonyl and the backbone of _A_I203. FR3 does not appear to make any distinct interactions in its position above the β9−β10 loop, but its proximity creates a situation where mutations, even in the FR, would cause steric clashes and destroy VHH affinity. _Nb_Y58 and the backbone carbonyl of _Nb_G57 from the CDR2 make additional polar interactions with the β9−β10 loop of the principal face.

VHH15 and the version solved in the structure (VHH15S) differ with mutations: A55T and A56T (in CDR2), as well as L32I, R33V, and D35R (just after CDR1). Only L32I is in close proximity, and the others are solvent facing mutations. The reported sequence of VHH4 is even more similar to VHH15S, and therefore it would be assumed that it binds to the same resolved location. Its sequence differs from VHH15S with the same A55T and A56T mutations that were described as showing no functional effect between VHH15 and VHH15S, as well as mutations in FR2: E44Q, CDR3: A104I, and just before CDR1: Y24A. The FR2 mutation would clearly also have no effect, and it is not evident how either Y24A or A104I produce a PAM effect upon VHH15S. A104I is a structural mutation that could have implications on the conformation of the CDR3. Structural analysis is needed to see what conformational changes this mutation may imply (Figure 6B.). The mutation Y24A simply removes a steric hindrance and would allow for I203 of the β9−β10 loop to bury deeper, but it is unclear how this would convert an inhibitor to a PAM because the interactions with the CDR1 would remain the same and this mutation would rather pull out the β9−β10 loop than close it in tighter, an aspect more commonly associated with antagonism.^1^ The removal of the steric hindrance could potentially also affect the conformation of CDR1, but again structural validation is needed.

In addition to these described VHHs, six more VHHs were described against the 5-HT_3_ABR, where, of the two VHHs for which functional tests were reported, both VHH5_AB_ and VHH13_AB_ act as agonists, but in binding they were described as showing no specificity between the 5-HT_3_A- and AB-Rs.^38^ Being selective for the A–A subunit interface would not preclude binding to the 5-HT_3_ABR, as the A–A interface is also present in 5-HT_3_ABR.^49^ The differences between VHH15S and VHH5_AB_ are extensive, having only three, one, and one residues in common throughout CDRs 1, 2, and 3, respectively, but the mutations and extension of the CDR3, along with the shorter CDR2 (Figure 3), do not appear to preclude binding to the same location as VHH15S, however, _Nb_S27Y in CDR1 may impede VHH5_AB_ binding in the same location or be the major cause of functional reversal between VHH15S and VHH5_AB_. Moreover, the mutations and extension of the CDR3, along with the reduction of CDR1 in VHH13_AB_, ensure that its binding, and possibly overall location, must be different than VHH15S. Therefore, it is difficult to assess either VHH’s mechanism of action without more information. The remaining VHHs did not have functional data reported but were evaluated for specificity, and the data conveyed that VHH9_AB_ was specific for the 5-HT_3_ABR, VHH10_AB_ was in general specific but a very poor binder to the 5-HT_3_ABR, whereas VHH18_AB_ and 21_AB_ had a preference for the 5-HT_3_ABR but were not fully specific.^38^

Unfortunately, a detailed characterization, which considers their full functional activity, selectivity, and binding location, is not described for any of the VHHs. This makes it difficult to properly evaluate all of them.

### 5.2 nAChR

**Figure 8:**
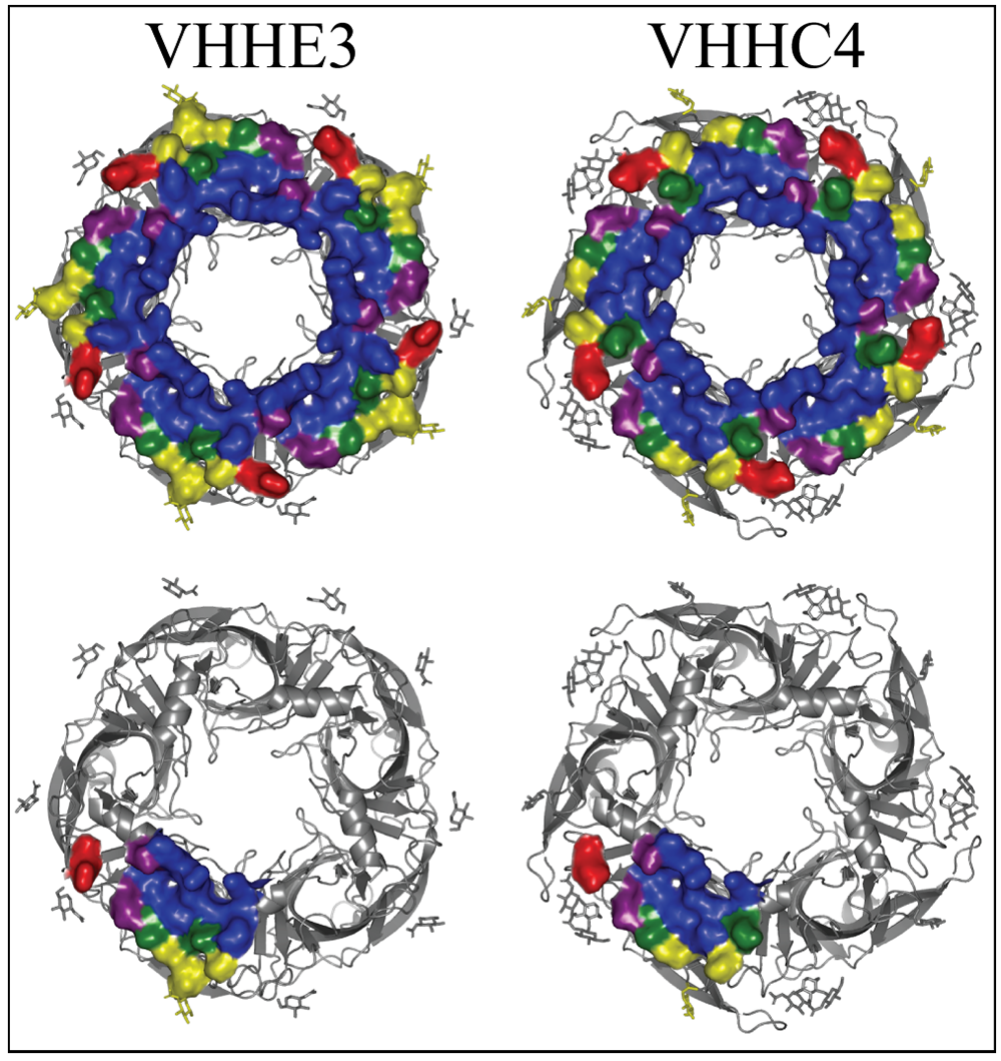
VHH binding surface on the α7-nAChR. Top down view of α7-nAChR-ECD structures with bound VHH name above the structures and color-coded surface representation of residues within 4 Å of this VHH following the same color code as Figure 4. Five VHHs are bound to the pentamer. For clarity a single VHH binding site is denoted for each below the fully bound representation. PDBs (left/right): 8ce4/8c9x.

**Figure 9:**
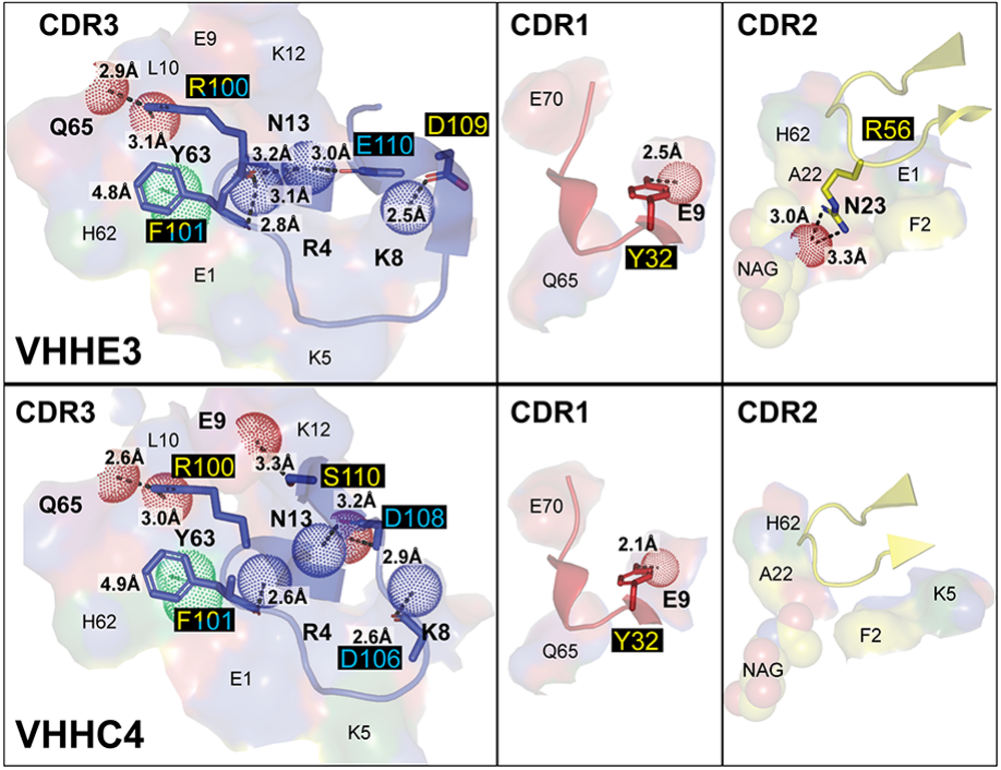
VHHE3 and VHHC4 pharmacophore interactions. Detailed interactions of each CDR from VHHE3 (top) and VHHC4 (bottom) with the α7-nAChR interface depicted in Figure 8. CDR3 makes the principal component of binding to the apical main immunogenic region, whereas CDR2 contains the functionally distinguishing A56R variation. Each panel, labeled by its respective CDR, is depicted, color coded, and labeled as in Figure 5, with the addition that aromatic side chain interactions are shown with a green dashed line and the distance indicated, and aromatic interacting atoms from the subunit residues are depicted as green dots.

Currently, only the α7-nAChR has described VHHs, whereas Fabs have been developed against the α3β4-^50^ and α4β2-nAChRs.^51^ The VHHs against the α7-nAChR were created in a manner different than the aforementioned VHHs, in that rather than using purified protein, whole-cells expressing the protein were injected into alpacas and used as the antigen to create desired antibodies.^39^ Seven unique sequences were obtained from this reported library, and only two of these were described as displaying specific binding to the α7-nAChR. Both of these VHHs were reported to show selectivity over other predominant neuronal nAChRs (α4β2- and α3β4-). Functional characterization showed that VHHE3 is a weakly binding moderate PAM (100 nM range) that easily washes out after 5 min. VHHC4 was reported to compete with VHHE3 but showed no modulation of the receptor activity up to the low μM range. Both VHHs bind in exactly the same location: apically, against the main immunogenic region (MIR) of the α7-nAChR, with CDR3 mainly anchoring the Nb and CDR2 involved in its functional modulation (Figure 8, PDBs 8ce4 and 8c9x). For both VHHE3 and VHHC4, _Nb_R100 of the CDR3 makes a polar interaction with the carbonyl backbones of _α7_Q65 and _α7_Y63 in the MIR, whereas _α7_R4, from the α1-helix of the complementary subunit, makes a polar interaction with the backbone carbonyl of _Nb_F101. _Nb_F101 also makes a coordinated Y-π-stacking with _Nb_W53 of the CDR2, and _α7_Y63 from the MIR (Figure 9). With proper refinement, the very close proximity of _Nb_Y32, from the CDR1, with _α7_E9, from the principal α1-helix, would increase to show an appropriate polar interaction. Meanwhile _α7_K8 from the complementary α1-helix makes a polar interaction with _Nb_D109 of the CDR3 in VHHE3, whereas because of the shorter CDR3 in VHHC4, this interaction changes to be with the carbonyl backbone of _Nb_D106. The two VHHs differ in binding where _Nb_R100’s backbone carbonyl, in VHHE3, makes polar interactions with the side chains of _α7_R4 and _α7_N13, whereas in VHHC4 these interactions are much weaker, interacting at a 3.5 Å distance. Additionally, _α7_K8 from the complementary α1-helix makes a polar interaction with _Nb_D109 of the CDR3 in VHHE3, whereas because of the shorter CDR3 in VHHC4, this interaction changes to be with the carbonyl backbone of _Nb_D106. Meanwhile, _α7_N13 at the end of the α1-helix of the principal α7-subunit makes polar interactions with the backbone of _Nb_D108 in the CDR3 of VHHC4, where the interaction with the backbone carbonyl is maintained with _Nb_E110 in the CDR3 of VHHE3. VHHC4 has an additional polar interaction through _Nb_S110 with the side chain _α7_E9. VHHE3 has a unique polar interaction from _Nb_R56 of the CDR2 with the glycosylated _α7_N23 in the MIR, and indeed this _Nb_R56A is the main distinguishable mutant that converts the PAM activity of VHHE3 to no perceived modulation by VHHC4.

### 5.3 ELIC

Eight reported nanobody families were isolated against a prokaryotic Erwinia ligand-gated ion channel (ELIC) found in *Erwinia chrysanthemi*.^52^ They have been shown to modulate channel activity in both a positive or negative manner, and some even act as a silent binder.^53^ This prokaryotic receptor is thought to belong to a pLGIC receptor subfamily unique to prokaryotes,^2^ therefore the specific functional relevance of each VHH may not be pertinent, but it would be appropriate to discuss potential binding locations noting the VHH’s general function. From the structurally resolved nanobodies, three different binding regions to the ELIC ECD may be observed.

**Figure 10:**
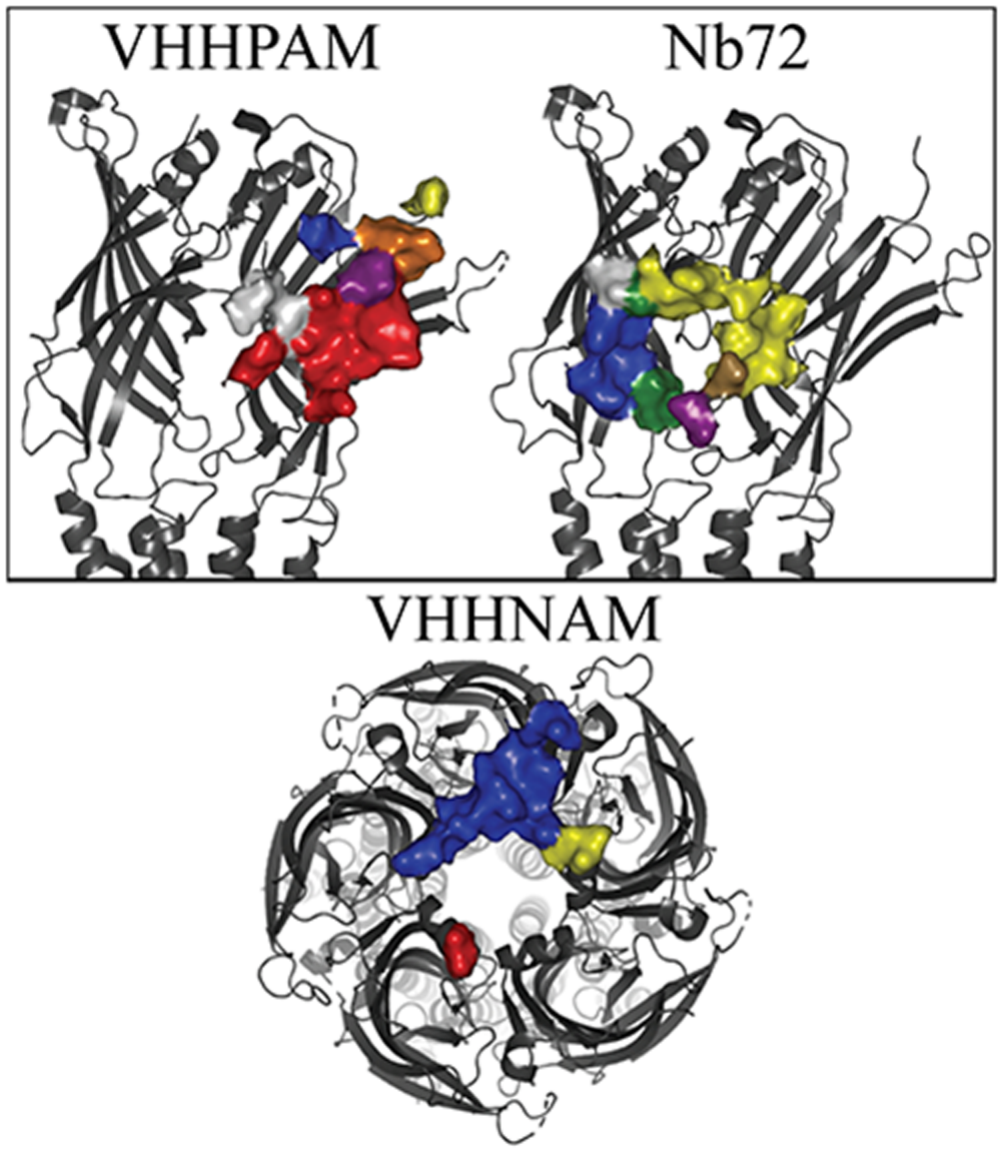
VHH binding surface on ELIC. Color-coded surface representation of residues within 4 Å of the bound VHH follows the same color code as Figure 4. Top row: Side view, cartoon representation, of two subunits involved in the subunit-interface binding, zoomed in on the ECD, with the bound VHH name listed above the structure. PDBs (left/right): 6ssi/6hjy. Bottom: Top-down view of ELIC. Only one VHH is bound per pentamer. PDB 6ssp.

The PAM nanobody, with the exception of _Nb_Q1 making a polar interaction with the backbone of D176, makes interactions with a single ELIC subunit, which arise primarily through its CDR1 (Figure 10, PDB 6ssi). _Nb_N28 makes a polar interaction with the backbone of A166, and starting with its backbone carbonyl up until the backbone of _Nb_N32, the CDR1 has an antiparallel β-sheet interaction with that of strand β8, where _Nb_N32 anchors this with an additional polar interaction with the backbone carbonyl of F142. The backbone of _Nb_I33 also interacts with the carbonyl of S143 in β8. The VHH’s short CDR3 makes no perceivable interactions, and its CDR2 makes a single polar interaction between _Nb_Q50 and R141 from strand β8.

The inhibiting nanobody (Nb72) was shown to bind below the ligand binding site and makes most of its contacts through the CDR2 (Figure 10, PDB 6hjy). From _Nb_G56 to _Nb_Y60, the CDR2 of this Nb also forms an antiparallel β-sheet interaction, this time with strand β9. R174 of strand β9 also makes a polar interaction with the backbone carbonyl of _Nb_F68 and side chain of _Nb_T69 from the FR3. In addition, the backbone carbonyls of _Nb_G52 and _Nb_R53 make polar interactions with the complementary N151, on the β8−β9 loop, and its backbone, respectively. _Nb_G55 also makes an interaction with its backbone to the carbonyl backbone of T149. Finally, _Nb_R53 buries deep enough to form a salt bridge with D113 of the Pro Loop (equivalent of the Cys-loop in mammals). CDR1 mainly points toward the solvent, yet has a lone weak polar interaction near the end with _Nb_N32 and the carbonyl backbone of I152 of the β8−β9 loop. Whereas, the CDR3 makes extensive contacts near the ECD–TMD interface with the Pro loop and the β10 sheet, specifically: _Nb_D104 with H169 and R194, _Nb_N110 and the backbone of _Nb_S103 with Q125, _Nb_S103 with N112, and the backbone carbonyl of Q125. In addition, _Nb_R99 forms a salt bridge with D153 of the complementary β8−β9 loop.

The other inhibiting nanobody acts as a NAM on ELIC and was reported to bind to the top of the receptor (Figure 10, PDB 6ssp). It sits on top of one subunit (*n*), making extensive contacts through its CDR3 with the apical pore-lining α-helix located between sheets β4−β5. The hydrophobic residues of the α-helix in the CDR3 (Figure 3) bury themselves near W66, meanwhile _Nb_D103, at the beginning of this helix, forms a salt bridge with R65, and its backbone makes a polar interaction with N69, which also weakly interacts with the backbone of _Nb_D101. N69’s carbonyl backbone in turn makes a polar interaction with _Nb_S112. The CDR3 also makes contacts with the complementary, *n*+1, subunit through _Nb_D101 and _Nb_D117 both forming salt bridges with R65_*n*+1_, as well as polar interactions between _Nb_Y114 and Q62_*n*+1_, and weakly between _Nb_Y118 and N69_*n*+1_. CDRs 1 and 2 occlude the pore, where CDR2 is within 4 Å of the *n*–1 subunit’s α-helix and CDR1 is within 4 Å of the *n*+2 subunit’s α-helix.

The binding location of the reported silent nanobodies has not been assessed, nor have any of them been structurally resolved, therefore it is not possible to evaluate their interactions with the receptor.

## 6 Modifications of VHHs

One of the biggest advantages of nanobodies is their single-chain structure and small size. That enables easy introduction of various types of modifications, which significantly expands the possibilities of uses for VHHs. Genetic engineering and/or chemical modifications of nanobodies may result in the improvement of some of their properties or emergence of new ones.^54^ The following techniques may enhance their use as tools for structural resolution attempts, functional studies, as well as their direct use in *in vivo* applications, e.g., as therapeutic agents, with respect to pLGICs.

### 6.1 Humanized Nbs

Although Nbs are characterized by low immunogenicity, their humanization may be necessary to create biocompatible tools for *in vivo* treatment. This is to minimalize the probability of an undesirable reaction from the immune system after application.^26,40^ The humanization strategies are based on the introduction of specific genetic mutations into the nanobody sequence. Mutagenesis most often involves FRs that lead to maximal mimicking of the human sequences.^55^ Changes mainly concern amino acids located on the surface of the molecule (solvent exposed), however, other residues may also be exchanged. The general goal is to maximize the replacement of amino acids with those found in human Abs, which still allows for maintaining the full properties of the Nb.^56^ However, too extended modifications can greatly decrease the stability of a VHH and lead to a reduction of antigen affinity.^31^

### 6.2 Protein Fused Nbs

#### 6.2.1 Nb–Nb Fusion Proteins

Nanobodies can be combined together in various ways. The molecules are joined using a specific flexible linker, usually composed of repeating, short sequences of glycine and serine residues. Among nanobody fusion proteins, a few variants may be distinguished:

##### Multivalent Nanobodies

**Figure 11:**
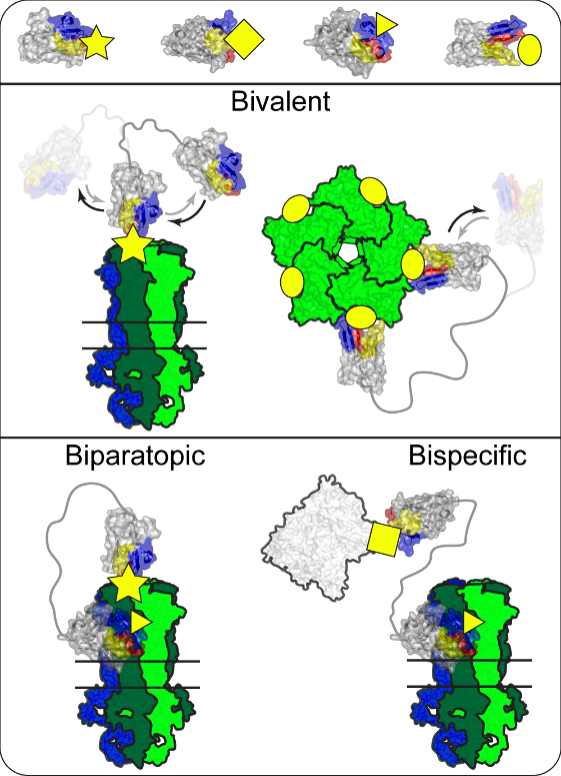
Nb–Nb fusions. Examples of Nb–Nb fusion types using VHHs (top row) that bind to different epitopes (yellow symbols). Example of two types of bivalent molecules (middle row), where identical VHHs are fused together. Left: Binding to a unique, star, epitope on a receptor. Higher affinity is achieved through association of the linked VHH, which was already in close proximity, when the bound VHH dissociates (black arrows) and vice versa (gray arrows, faded VHH). Right: Top-down view. Binding to an, oval, epitope that occurs five times on a homopentameric receptor. An increased affinity is achieved from the rapid reassociation (gray arrow, faded VHH) of a single dissociated VHH (black arrow), during which the other linked VHH stays associated. Biparatopic VHH fusion (bottom row, left) is similar to this but the fusion consists of two unique VHHs that bind to different epitopes on the same receptor. Right: Bispecific VHH fusion consists of two unique VHHs binding to two unique proteins (pLGIC and albumin [white]).

Nbs may be combined into a larger unit, such as multivalents, composed of two to several Nbs recognizing the same epitope. These constructs are characterized by improved binding affinity as compared to the single Nb. This is achieved through keeping the unbound Nb in close proximity to allow for rapid association upon dissociation of the bound Nb in the case of a single epitope on a given protein (Figure 11, bivalent left). In the case of homo-oligomeric structures that contain the same epitope multiple times, it may be achieved from the rapid reassociation after dissociation of the same Nb as a result of being linked to the bound Nb (Figure 11, bivalent right).

Since 2010, a number of multivalent Nbs were created and characterized as potential therapeutics for cancer, autoimmune diseases, or infectious diseases. Several of these have started clinical trials (phases I, II, and III), demonstrating that multivalent Nbs are a promising tool in the effective treatment of many diseases.^57^

The bivalent NbE3-E3, against α7-nAChR, was created by connecting the C-terminal of one VHHE3 and N-terminal of the second VHHE3 using a linker composed of the sequence GGGGS repeated four times. This construct showed a significantly increased affinity for the α7-nAChR, when compared to a single VHHE3, yet did not lose the PAM behavior and even exhibited an irreversible binder property.^39^

##### Biparatopic Nanobodies

Whereas the aforementioned bivalent Nbs can greatly increase the antigen binding affinity through two molecules recognizing the same epitope, in contrast, biparatopic Nbs are constructs of two molecules recognizing different antigens on the same target molecule (Figure 11). Biparatopicity diversifies a Nb’s antigen profile, thereby significantly increasing the overall affinity for the individual target molecule.

##### Bispecific Nanobodies

Bispecific Nbs are the result of the fusion of two VHHs which bind to different antigens on different molecules (Figure 11). This achieves a multispecificity of the Nb, which may help increase their efficacy by reducing their clearance time from the plasma. Bispecificity may either diversify a Nb’s antigen profile or narrow its specificity through targeted localization.

#### 6.2.2 Fc-Conjugated Nbs

An Fc-conjugated Nb is a fusion protein composed of the VHH domain connected with the Fc region of a conventional antibody (mostly IgG type). The Fc domain is naturally responsible for the activation of immune system cells during infection.^58,59^ The fact that VHHs are the HCAbs without the FC domain limits their effectiveness, and reduce their prospects in immunotherapeutic applications. Fc conjugation may extend the Nb’s biological properties to include effector functions of this domain.^37,60−62^ The bigger size of such construct may also increase the serum half-life of a Nb, making it more suitable for some *in vivo* applications. Additionally, fusion with an Fc domain may promote dimerization of the Nb molecule, acting the same way as a linker, and enabling the creation of bivalent, bispecific, or biparatopic variants of Nbs which may enhance antigen binding affinity.^39^

Human Fc-domain-linked constructs of the aforementioned α7-nAChR Nbs, VHHC4 and VHHE3, were created using a 21-residue C-terminal linker which contains two cysteine residues that facilitates dimerization of the created chimeras.^39^ Such constructs may not only be used to enhance immunostaining of weak binders, but also as immunotherapeutic agents for passive immunization.^63^

#### 6.2.3 Megabodies (Mbs)

**Figure 12:**
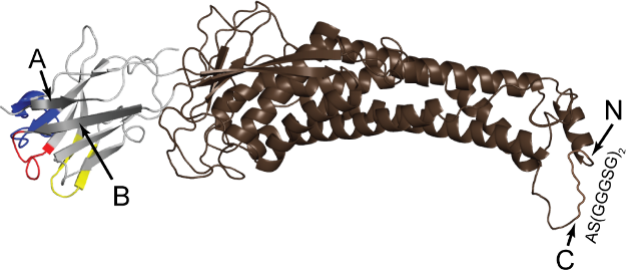
MegaBody38 with c7HopQ. CDRs 1, 2, and 3 are color coded red, yellow, and blue, respectively on Nb38 (from PDB 6i53). β-sheets A and B (darker) on Nb38 are labeled, as well as linker-connected (and truncated) N- and C-termini of HopQ (brown, from PDB 6qd6).

Mbs are chimeric proteins composed of a Nb fused to scaffold proteins.^46,64,65^ In these constructs, the scaffold protein is attached to the loop between β-strands A and B of the Nb’s FR1 (Figure 12). Such specific positioning of the scaffold protein in the Mb construct not only increases the size of molecule, but also makes the entire structure stiffer while maintaining binding affinity and specificity. Mbs seem to be a great innovative tool used to overcome the size limitation problems that occur in cryo-electron microscopy analysis.

The aforementioned Mb38, or MegaBody38 (Mb_Nb38_^cHopQ^), is a fusion protein of Nb38, and the *H. pylori* outer membrane adhesin protein HopQ, as a scaffold protein. The proteins were connected into a single protein chain through intramolecular linking between β-strands: A/B of Nb38 and S3/S4 of HopQ. Additionally, the N- and C-termini of the HopQ were connected with a flexible glycine-rich linker to close the whole sequence.^46,65^ This Mb was created and used to increase the quality of cryo-electron microscopy images of α1β3γ2L–GABA_A_R. The combined properties of this Mb, including the rigid fusion, larger size (∼58 kDa), and intrinsic properties of the scaffold protein, while maintaining full α1-subunit specificity, allowed for various angles of the receptor, in a more even distribution as compared to Nb38 alone, to be captured, which significantly facilitated particle alignment.^46,64^ However, as previously mentioned, the complex of Mb38 with α1β3γ2L–GABA_A_R showed only one Mb bound to the interface of α1/β3 subunits, compared to the reported ability of Nb38 to bind both α1/β3 and α1/γ2 ECD regions, albeit at significantly different concentrations.^45,46^

Other Mbs were created based on Nb25, which selectively recognizes the β3-subunit of GABA_A_Rs.^65^ These two Mbs differ in the scaffold protein: (1) *H. pylori* outer membrane adhesin protein HopQ (Mb_Nb25_^c7HopQ^), and (2) *E. coli* K12 glucosidase YgjK (Mb_Nb25_^cYgjKE2^). The general design of these fusion proteins is analogous to Mb38. Nb25 was connected with the scaffold protein through S3/S4 β-strands of HopQ, or A′S1/A′S2 β-strands of YgjK. These two constructs showed that the choice of the scaffold protein is very important and may affect the Nb properties. Structural studies of β3–GABA_A_R showed that the presence of Mb_Nb25_^c7HopQ^ resulted in obtaining different orientations of the receptor structure, similarly to Mb38. However, Mb_Nb25_^cYgjKE2^ bound to β3–GABA_A_R led to only one receptor orientation: the top view, which, when compared to the described orientation of the receptor alone or bound to Nb25, was worse.^65^

### 6.3 Chemically Modified Nbs

Nbs can be modified with various chemical components, resulting in the use of a single VHH (recognizing one, specific epitope) in many different types of analyses/techniques. Such chemical conjugation can be achieved using *N*-hydroxysuccinimide (NHS) ester groups or maleimide groups that interact with primary amines or thiol groups in the nanobody sequence, respectively.^39,66^ While the labeling, with the use of NHS esters, is nonselective and leads to creation of heterologous population of modified Nbs, maleimide coupling may be selective, if a single, unpaired cysteine is present in the protein chain, naturally or added as a result of genetic manipulation, and the coupling is performed at the proper chemoselective pH. Site-selective conjugation may be also achieved with the use of non-natural amino acids incorporated into the Nb sequence^67^ with click-chemistry techniques.^68^ Due to the structural arrangement of Nbs (Figure 2), the labeling is usually carried out at the C-terminus to avoid interference with the antigen binding surface.

#### 6.3.1 Imaging with Fluorescent Coupled Nbs

Fluorescent labeling is a common coupling of Nbs through a direct connection with a fluorescent dye. The relative ease of labeling, and creation of such Nbs, renders them effective tools for various fluorescence microscopy techniques and assays, including Förster resonance energy transfer (FRET)-based assays, as well as immunochemical methods, such as Western blotting and ELISA.

For example, VHHE3, recognizing the α7-nAChR, was labeled with Alexa Fluor 488, where the C-terminal of the Nb was extended by a 15-residue, flexible linker, (GGGGS)_3_, followed by a CSA motif, allowing for direct connection of a maleimide-linked fluorescent dye to the thiol group of the cysteine residue. This allowed for direct evaluation of the Nb in question through immunofluorescent assays.^39^

Fluorescently fused, or even chemically labeled, Nbs against pLGICs or tags added to the receptor for purification purposes may also be used to evaluate receptor stability and expression during a purification process. For example, a green fluorescent protein (GFP)-fused Nb was used as a replacement for a fluorescent-protein-fused receptor in the fluorescence size-exclusion chromatography methodology,^69^ in order to screen the expression stability of ZAC orthologues.^70^ The ZAC included a C-terminal ALFA tag (SRLEEELRRRLTE, an α-helical, hydrophilic motif, with a net neutral charge that is devoid of amine-reactive residues^71^), for which the GFP-Nb used is selective. Fluorescent compounds linked chemically may enhance the fluorescent protein catalogue to allow for a diverse range of fluorophores to choose from in experiments related to pLGICs.

#### 6.3.2 Other Click-Chemistry Coupling

Although simple and widely used, maleimide-based coupling is not perfect and has some drawbacks. It has been documented that thiosuccinimide linkage may be unstable *in vitro* due to the retro-Michael type reaction (a thiol exchange with other reactive thiol groups, such as free cysteines, glutathione, or albumin in the plasma).^72,73^ This is especially important in the case of Nb-based drug delivery systems. Uncontrolled release of drug molecules in unintended locations may have a huge impact on its effectiveness, and more importantly on patient safety, having adverse effects on healthy cells/tissues. The stability of a thiosuccinimide linkage is largely dependent on the location of the cysteine in the protein chain (hidden or solvent exposed) and the pH of the environment.^74,75^ There are methods to ensure the stability of the succinimide–thioether ring, such as specific hydrolysis after the reaction with the thiol group, which has been shown to significantly increase *in vitro* stability.^73^

**Figure 13:**
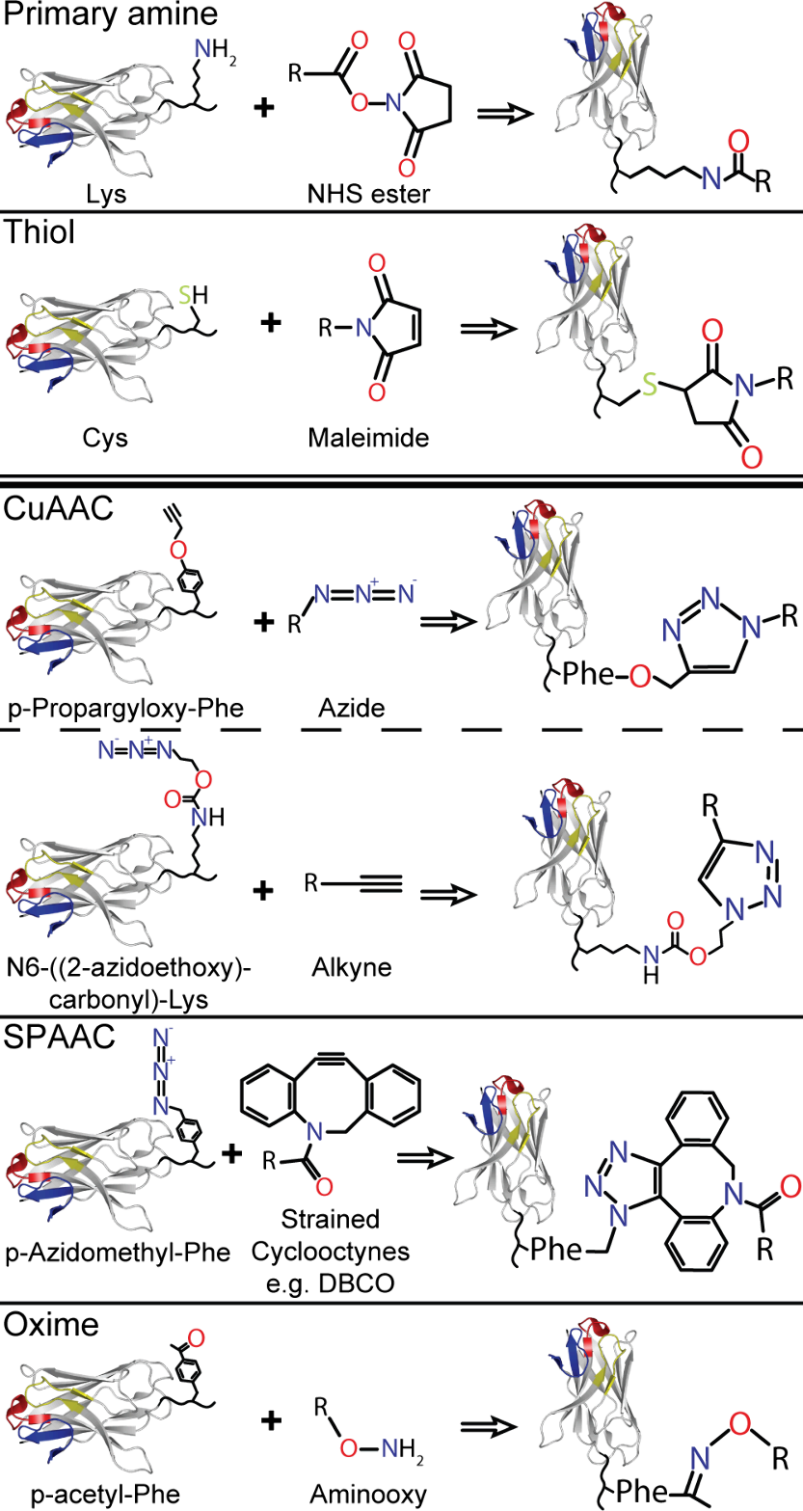
Chemical conjugation methods of Nbs. Options for chemical conjugations include conjugation to naturally occurring functional groups (above double line), such as primary amine and thiol groups, or non-natural amino acid incorporation (below double line, with some of the common non-natural moieties represented). These incorporated amino acids are displayed on a C-terminal Nb linker, with the Nb CDRs color coded for orientation. The bio-orthogonal pair is listed and shown in the middle with the corresponding Nb conjugation product on the right after the arrow.

Other click-chemistry reactions (Figure 13) may achieve site-homogeneous synthesis with a more stable linkage that remains intact in *in vivo* conditions. These reactions consist of bio-orthogonal reactive pairs, such as carbonyl–aminooxy (yielding an oxime product linker) or azide–alkyne (yielding a triazole product linker), producing a high yield, requiring mild reaction conditions, and have fast reaction rates.^76^ A reactive group from the bio-orthogonal reactive pair: carbonyl or azide, and aminooxy or alkyne, must be introduced to the Nb, while the corresponding group introduced to the drug molecule.^77^ Azide–alkyne cycloaddition reactions may be copper catalyzed (CuAAC), or to avoid the disadvantage of Cu(I) ion toxicity that generates reactive oxygen species, the reaction may be achieved using strain-promoted cycloaddition (SPAAC), albeit at much slower reaction rate in comparison to copper catalysis. SPAAC may be achieved with strained cyclooctyne moieties such as cyclooctyne, bicyclononyne (BCN), dibenzoannulated cyclooctyne (DIBO), dibenzocyclooctyne (DBCO), or aza-dibenzocyclooctyne (DIBAC), among others.^76,78^ Oxime click reactions are also a viable alternative to copper-catalyzed azide–alkyne reactions, but likewise are significantly slower. However, with the use of certain catalysts this reaction rate may be increased, albeit with the reintroduction of a toxic catalyst in some cases.^79^ The use of toxic catalysts are important considerations if coupling is intended to occur *in vivo*, or *in vitro* in the presence of cells.

#### 6.3.3 Chemically Coupled Nbs for Drug Delivery

These discussed click-chemistry methods seem to be a perfect tool for the creation of Nb-drug delivery systems. High-antigen affinity, small size (allowing for easy tissue penetration), and reduced immunogenicity of Nbs, together with strong, irreversible, site-specific conjugation with a druggable molecule, may result in the development of innovative targeted therapy. Due to the simplicity of Nb production with either an added cysteine or lysine, or simply using such existing solvent accessible residues, maleimide or NHS ester linkage are frequently used for drug coupling. However, incorporation of unnatural amino acids such as *p*-acetyl-l-phenylalanine, *p*-azido-l-phenylalanine, *p*-azidomethyl-l-phenylalanine, l-azidohomoalanine, *N*6-((2-azidoethoxy)carbonyl)-l-lysine, l-homopropargylglycine, and *p*-propargyloxy-l-phenylalanine may be used to obtain carbonyl–aminooxy, or azide–alkyne linkage (Figure 13).^80^ The choice of click-chemistry method, and more importantly the linker obtained as a product, must be evaluated for each specific use, as the product may lead to unwanted immunogenicity.^81^ In addition to the choice of nanobody linkage, many options exist for the linker itself depending on the desired action of the drug. These options, including creating a cleavable linker, or having the attached drug caged, have broadly been discussed for Abs, and the principles are the same for Nbs.^82^ Nbs as drug delivery agents in chemotherapy have been extensively reviewed, and some of the same tenets may be applicable to pLGICs.^41,43,80,83^

#### 6.3.4 Opto-Nbs

**Figure 14:**
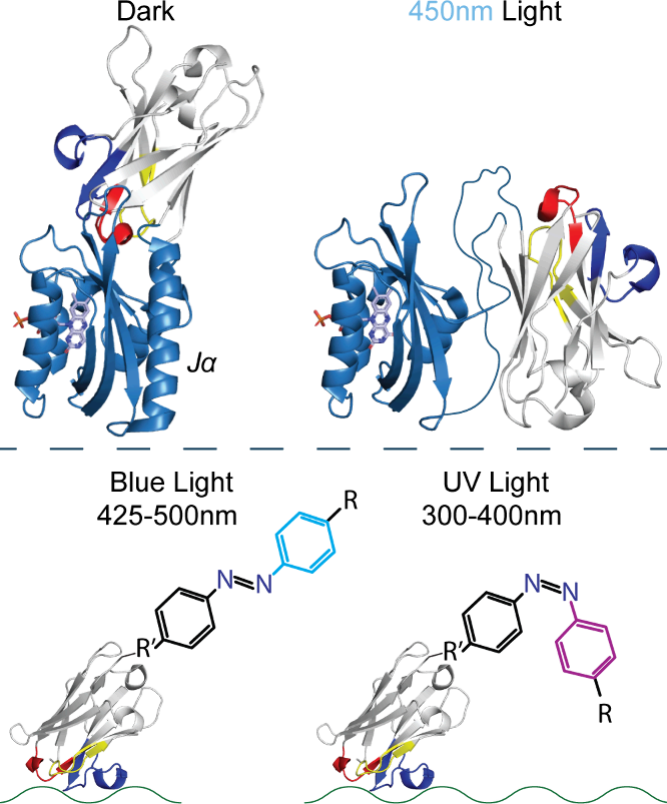
Opto-Nb constructions. Photoactivation may modify the Nb (model from PDB 8ce4 with CDRs 1, 2, and 3 colored red, yellow, and blue respectively) activity directly (top) or may modify a chemically attached photoreactive ligand (bottom). Top: Truncated *A.s.*-LOV2^85^ (modified from PDB 2v0u, colored sky-blue with flavin mononucleotide, light-blue, displayed in stick representation) inserted between _Nb_A74 and _Nb_K75, shown in dark/inactivated (left) form or light/activated (right) form, where the Jα helix undocks and loses structure adding flexibility.^87^ Bottom: Azo-benzene moiety, chemically linked (R′) using any of the aforementioned chemical modifications in this Perspective with rigid linkers that may be used to allow for a ligand (R) to interact with a Nb target protein (represented by the green wavy line) after conformational change under UV light.

Photoswitchable molecules have already been used to study the mechanics of pLGICs.^84^ Photoswitchable nanobodies could enhance experimental use of optogenetics, where pLGIC silent binding Nbs could be converted to opto-nanobodies.^85^ Either known antagonists, including pore-blockers, or agonists may be coupled at the end of a suitable length linker, which contains a photoswitchable element, such as an azo-benzene, using the aforementioned chemical modification methods. Additionally, the Nbs themselves may also be light-activated through the insertion of a photoswitchable domain, such as the LOV2 of *Avena sativa* (*A.s.*-LOV2),^85^ or through the incorporation of photocaged amino acids into the Nb sequence (Figure 14).^86^ These Nbs could be used as tools to further study specific functional transitions of pLGICs, with high precision.

### 6.4 BBB Penetrating Nbs

Crossing the BBB is crucial for the studying, or treatment, of nervous system disorders. It has been mentioned earlier that some Nbs can cross the BBB. Although this ability is based mostly on the natural properties of Nbs that enable them to interact with BBB elements, crossing of the BBB by Nbs may be limited by the negative charge on their surface. One example to remedy this is to change the overall surface charge via fusion with an enhanced GFP.^88^ As a result of the fusion, the intrinsic pI increases and leads to a positive charge of the chimera in the plasma pH environment. This enables interaction with the endothelial cell membrane and thus crossing of the BBB through adsorptive-mediated transcytosis. Moreover, direct protein coupling of cell-penetrating peptides may serve as transporters, masking the presence of the VHH, to allow for BBB crossing.^89^ Appropriately modified Nbs recognizing pLGICs could be a useful tool in imaging of receptors and studying their distribution in the physiological and pathological central nervous system. Moreover, they could serve as drug-delivery agents in neurological disorders in which pLGICs are implicated.

### 6.5 Nb-Base Imaging via Positron Emission Tomography (PET)

PET imaging allows for a noninvasive (compared to biopsy) method of obtaining information on biological processes occurring at the cellular and molecular level. However, the specificity of commonly used small molecules, such as [^18^F] fluorodeoxyglucose (FDG) or [^18^F] fluorothymidine (FLT), is questionable and can lead to false-negative or false-positive results due to the heterogeneity of many diseases at the molecular level. Radiolabeling of Nbs seems to be an interesting tracer alternative in PET techniques.^90^ High specificity of Nbs toward antigens may allow for precise molecule/cell targeting and the development of personalized treatments for nonresponding patients.

Nbs may be conjugated with the radioactive molecule directly (into Tyr or His side chains), or indirectly via a specific prosthetic group [auxiliary groups, e.g., *N*-succinimidyl-3-(4-hydroxyphenyl)propionate, *N*-succinimidyl-3-iodobenzoate (SIB), *N*-succinimidyl-5-iodo-3-pyridinecarboxylate (SIPC), or *N*-succinimidyl 4-guanidinomethyl-3-iodobenzoate (SGMIB)]^91^ or a bifunctional chelating agent (e.g., diethylenetriaminepentaacetic acid (DTPA), 1,4,7,10-tetraazacyclododecane-tetraacetic acid (DOTA), 1,4,7-triazacyclononane-triacetic acid (NOTA), hexa-histidine (His-tag), and 6-hydrazinonicotinic acid (HYNIC)).^91,92^ The most common isotopes used for protein labeling are ^125^I, ^123^I, ^124^I, ^131^I, ^18^F, ^68^Ga, ^111^In, and ^99m^Tc.^91^

## 7 Discussion

Due to their size and ease of production, Nbs were targeted and developed against pLGICs as tools to help structural resolution, first, in crystal-packing, and subsequently, in subunit identification and protein orientation for cryo-electron microscopy. Of these VHHs, the Nb binding locations varied, but each human pLGIC discussed herein had multiple Nbs reported, with varying functional effects that bind to a similar location. Overall, these identified allosteric sites mimic the behavior of the endogenous neurotransmitter binding-site of pLGICs, in that, depending on the ligand’s (in this case Nb’s) pharmacological properties, receptor activation or inhibition may occur. In the case of the GABA_A_Rs, drastically different Nbs were found to bind peripherally to the ligand-binding site just below the β9−β10 loop, binding predominantly to the principal subunit. The differences in Nb sequence created unique stoichiometric affinities, as well as different functional effects. Based on the subunits’ roles in GABA_A_R activation, it is conceivable that modification of the already described VHHs could maintain specificity but inverse the functional effects reported on the GABA_A_R subtype. Both the 5-HT_3_Rs and nAChRs corroborate this concept. In the case of 5-HT_3_Rs, the VHH localized above the β9−β10 loop, binding again peripherally to the ligand-binding site, but predominantly against the complementary subunit, with minor mutations of this VHH changing an inhibitory effect to a potentiating effect. Whereas, in the case of nAChRs, minor sequence differences remove a potentiating effect but maintain VHH affinity to the same binding location. It is conceivable that further mutations in this situation could produce an inhibitory effect on receptor function.

The Nbs against ELIC further enhance this argument that all allosteric sites retain the gamut of functional effects bore by the endogenous ligand-binding site. ELIC’s PAM- and NAM-Nbs provide an excellent example of this as they exhibit the opposite function on their respective binding sites than other previously identified small molecules that act as allosteric modulators of this channel.^53^ Specifically, the PAM-Nb binding site has been previously reported as a binding site for the CU2014 fragment that acts as a NAM. Similarly, the NAM-Nb and positively acting flurazepam share a common binding region.

With the perspective given on the various modifications of VHHs, it is important to convey that, although an excellent tool for structural resolution purposes, the development of VHHs against the pLGIC family should not be limited to this. The currently published research implies that VHHs have a strong potential in therapeutic development against the plethora of neurological disorders that afflict the pLGIC family. Unlike previous antibodies developed against peptidic fragments, almost all of the described nanobodies bind at a subunit interface and are specific for a unique homo- or heterocross-subunit interaction. These VHHs may either target unique stoichiometries of the receptor family or help distinguish between multiple stoichiometries that contain the same interface recognized by the Nb. For example, as noted for GABA_A_Rs, Nb25 bound either twice in α4β3δ−GABA_A_Rs, or three times in β3δ−GABA_A_Rs.^47^ Additionally, targeted modification of VHHs may be made using information from larger Fab regions developed for aide in structural resolution. For example, two inhibitory GABA_A_R Fab domains have been described and structurally resolved that recognize unique subunit interfaces; where Fab115 recognizes the interface between β2-α1 with one finger in the ligand-binding pocket, binding peripherally predominantly on the complementary _α1_β8−β9 loop (PDB 7t0w), and Fab175 recognizes the α1−γ2 interface binding perpendicularly well above the ligand-binding site interacting near the MIR interface (PDB 7t0z).^93^ The interactions reported may be used to develop smaller VHHs targeting the same interfaces, and with modification potentially reverse the functional effects. Finally, through the above-described VHH modifications, with specific attention to their chemical modification, there is also a strong potential for further *in vivo* studies involving receptor localization, migration, and turnover.

Given the initial structural focus for the development of nanobodies, they may also help stabilize the ICD and/or they may be used as tools to study regulatory-protein interactions with the ICD. All of the identified VHHs were generated through camelid immunization. There have been recent developments of synthetic VHH libraries, which potentially may improve Nb generation efforts.^94,95^ The use of synthetically created Nbs omits the immunization step, and therefore the target protein’s environment may be more readily controlled. This removes complications that may arise from target-protein stability issues in animal serum after immunization. Although synthetic libraries lower the probability to find a targeted VHH, it may be the only option for such sensitive proteins. Furthermore, with the discovery of more and more pLGIC VHHs, synthetic libraries may prove to be an efficient mechanism to rapidly produce new Nbs. Targeted libraries could be developed using a consensus sequences, such as that in Figure 3, to increase the chances of isolating VHH specific for pLGICs.

## Accession Codes

The data sets analyzed for this review can be found in the PDB, https://www.rcsb.org/.

## Abbreviations

5-HT: 5-hydroxytryptamine/serotonin
5-HT_3_R: type 3 serotonin receptor
Abs: antibodies
ACh: acetylcholine
BBB: blood–brain barrier
BCN: bicyclononyne
BRIL: apocytochrome b562RIL
CDR: complementarity determining region
CuAAC: copper catalyzed azide–alkyne cycloaddition
DBCO: dibenzocyclooctyne
DIBAC: aza-dibenzocyclooctyne
DIBO: dibenzoannulated cyclooctyne
DOTA: 1,4,7,10-tetraazacyclododecane-tetraacetic acid
DTPA: diethylenetriaminepentaacetic acid
ECD: extracellular domain
ELIC: Erwinia ligand-gated ion channel
Fab: fragment antigen-binding
FDG: fluorodeoxyglucose
FLT: fluorothymidine
FR: framework region
FRET: Förster resonance energy transfer
GABA: γ-aminobutyric acid
GABA_A_R: γ-aminobutyric A receptor
GFP: green fluorescent protein
Gly: glycine
GlyR: glycine receptor
HCAbs: heavy chain only antibodies
HYNIC: 6-hydrazinonicotinic acid
ICD: intracellular domain
IgNARs: immunoglobulin new antigen receptors
MIR: main immunogenic region
nAChR: nicotinic acetylcholine receptor
NAG: *N*-acetylglucosamine
NAM: negative allosteric modulator
Nb: nanobody
NHS: *N*-hydroxysuccinimide
NOTA: 1,4,7-triazacyclononane-triacetic acid
PAM: positive allosteric modulator
PDB: Protein Data Bank
PET: positron emission tomography
pLGIC: pentameric ligand-gated ion channels
scFvs: single chain variable fragments
sdAbs: single-domain antibodies
SGMIB: *N*-succinimidyl 4-guanidinomethyl-3-iodobenzoate
SIB: *N*-succinimidyl-3-iodobenzoate
SIPC: *N*-succinimidyl-5-iodo-3-pyridinecarboxylate
SPAAC: strain-promoted azide–alkyne cycloaddition
TMD: transmembrane domain
VHH: variable domain of a heavy chain only antibody
ZAC: zinc-activated ion channel
