# Corrections to “VHH Nanobody Versatility against Pentameric Ligand-Gated Ion Channels”

**DOI:** 10.1021/acs.jmedchem.4c02703

**Published:** 2025-06-03

**Authors:** Dorota Nemecz, Weronika Nowak, Ákos Nemecz

Page 8502 Section 2: Typographical errors not caught during copy edits should be corrected as such: a comma after “peripheral,” in the second sentence of the first paragraph. An o is missing from “*Drosophila melanogaster*” in the second to last sentence.

Page 8507 Section 4.1: Last paragraph a comma should be removed after _Nb_R101 in the sentence “These interactions consist of _Nb_R101 making a salt bridge with the neighboring subunit _β3_E182 on the β8−β9 loop, ...”

Section 4.2: A reference to existing Fabs was left out the section. It should read as follows (with the inclusion underlined and an inclusion of a new ref:^1^ “Due to the ease with which the GlyR may be structurally resolved, there has not been a drive for the development of nanobodies against the receptor, although Fabs have been used to resolve native conformations of the GlyR.^1^ As such no currently published nanobodies exist, but that does not preclude their potential as tools and selective pharmaco-agents targeting GlyRs.”

Page 8508 Section 5.2: Although the data reviewed in this Perspective of the structures of VHH-E3 and VHH-C4 with the α7-nAChR was properly referenced, a reference to the article^2^ which introduces these structures was inadvertently left out. The reference to Figure 8 should read “(Figure 8, PDBs: 8ce4 and 8c9x)^**2**^”.

Page 8509–8510 Section 5.3: Similarly to the above correction references to PDBs’ respective articles (referenced as 52 and 53 in the Perspective and refs 3 and 4 in this correction) should be added after “(Figure 10, PDB 6ssi)^**53**^” in the second paragraph, “(Figure 10, PDB 6hjy)^**52**^” of the third paragraph, and “(Figure 10, PDB 6ssp)^**53**^” of the fourth paragraph.

Page 8510 Section 6.2.1 Multivalent Nanobodies, first paragraph: A comma should be added in the sentence: “This is achieved through keeping the unbound Nb in close proximity to allow for rapid association upon dissociation of the bound Nb, in the case of a single epitope on a given protein (Figure 11, bivalent left).”

Page 8514 Section 7 Paragraph three: In the middle of the paragraph there is an inadvertent “e” on the word aid. The sentence should read “Additionally, targeted modification of VHHs may be made using information from larger Fab regions developed for aid in structural resolution.”

Paragraph four: In addition to those already referenced in the Perspective (94 and 95), two additional references to synthetic libraries [refs 5 and 6 of this correction] should be added, after the sentence: “There have been recent developments of synthetic VHH libraries, which potentially may improve Nb generation efforts.”
